# Integrating public preferences into national reimbursement decisions: a descriptive comparison of approaches in Belgium and New Zealand

**DOI:** 10.1186/s12913-020-05152-2

**Published:** 2020-04-25

**Authors:** Christine Leopold, Christine Y. Lu, Anita K. Wagner

**Affiliations:** Division of Health Policy and Insurance Research, Department of Population Medicine, Harvard Medical School and Harvard Pilgrim Health Care Institute, Landmark Center, 401 Park Drive Suite 401, Boston, MA 02215 USA

**Keywords:** Public preferences, Insurance coverage, Prescription drugs, Health care, Qualitative research

## Abstract

**Background:**

Public health care payer organizations face increasing pressures to make transparent and sustainable coverage decisions about ever more expensive prescription drugs, suggesting a need for public engagement in coverage decisions. However, little is known about countries’ approaches to integrating public preferences in existing funding decisions. The aim of this study was to describe how Belgium and New Zealand used deliberative processes to engage the public and to identify lessons learned from these countries’ approaches.

**Methods:**

To describe two countries’ deliberative processes, we first reviewed key country policy documents and then conducted semi-structured interviews with five leaders of the processes from Belgium and New Zealand. We assessed each country’s rationales for and approaches to engaging the public in pharmaceutical coverage decisions and identified lessons learned. We used qualitative content analysis of the interviews to describe key themes and subthemes.

**Results:**

In both countries, the national public payer organization initiated and led the process of integrating public preferences into national coverage decision making. Reimbursement criteria considered outdated and changing societal expectations prompted the change. Both countries chose a deliberative process of public engagement with a multi-year commitment of many stakeholders to develop new reimbursement processes. Both countries’ new reimbursement processes put a stronger emphasis on quality of life, the separation of individual versus societal perspectives, and the importance of final reimbursement decisions being taken in context rather than based largely on cost-effectiveness thresholds.

**Conclusions:**

To face the growing financial pressure of sustainable funding of medicines, Belgium’s and New Zealand’s public payers have developed processes to engage the public in defining the reimbursement system’s priorities. Although these countries differ in context and geographic location, they came up with overlapping lessons learnt which include the need for 1) political commitment to initiate change, 2) broad involvement of all stakeholders, and 3) commitment of all to engage in a long-term process. To evaluate these changes, further research is required to understand how coverage decisions in systems with and without public engagement differ.

## Background

Public pharmaceutical reimbursement agencies are under enormous pressure to evaluate and potentially restructure existing coverage decision making processes. Health care spending is rapidly increasing in many countries, largely due to increasing pharmaceutical spending on new medications that are becoming available at very high prices to treat cancers and other chronic conditions of aging populations [1, 2]. Highly-effective therapies that benefit large numbers of patients like the direct-acting antivirals for hepatitis C, rapidly-approved pharmaceuticals with uncertainty of effectiveness and safety like many new cancer therapies, and gene therapies for orphan diseases like spinal muscular atrophy that may benefit few require renewed discussions of the ethical dilemmas that arise around which treatments and services to cover, for whom, with how much patient cost-sharing, and at what opportunity costs to society [3]. Daniels and colleagues have argued that “resource allocation decisions in health care are rife with moral disagreement and a fair, deliberative process is necessary to establish the legitimacy and fairness of such decisions … the process must be public (fully transparent) about the grounds for its decisions; the decision must rest on reasons that stakeholders can agree are relevant; decisions should be revisable in light of new evidence and arguments; and there should be assurance through enforcement that these conditions are met*.”* [4–7]

Searching for ways to optimize public pharmaceutical reimbursement and to identify health system priorities, some countries are placing more emphasis on public engagement and on identifying patients’ and public preferences [2, 8–10]. One of the aims of public engagement is to better align coverage decision making processes including those based on health technology assessments with public values, needs and preferences [9, 11] and to legitimize final coverage decisions [12, 13]. The term “public” can refer to different forms of engagement of different stakeholders. As Degeling and colleagues described, “Patients, advocates or consumers, otherwise known as partisan groups, are often engaged as witnesses or experts on a specific matter whereas lay, non-partisan or disinterested citizens are more frequently asked to be involved in broader policy-making decisions.” [14] Depending on a country’s health system structure and institutional settings as well as its political, societal and cultural environment and the intended purpose of the public engagement in coverage decision making, countries have chosen different processes of engagement [15, 16], ranging from deliberative citizens’ juries to large population surveys using discrete choice experiments [17, 18].

Baltussen and colleagues suggested a public outreach framework for health policy decision-making, the so-called ‘evidence-informed deliberative process’, which includes six steps of public participation in policy making: 1) situational analysis, 2) formation of stakeholder panel, 3) identification of relevant criteria, 4) collection of evidence on criteria, 5) deliberation, and 6) recommendation for implementation [19–21]. This framework builds the underlying concept of the present study.

While the concept of public engagement in health policy decisions is gaining more attention, little information is shared between countries or in the peer reviewed literature on countries’ processes and experiences of engaging the public in coverage decisions [22]. We describe the approaches of two countries, Belgium and New Zealand, toward adjusting existing reimbursement processes to account for public preferences. Both countries’ intent for restructuring the reimbursement system was to engage citizens to integrate their preferences in coverage decision-making through an evidence-informed deliberative process. The specific objectives of this study were to 1) investigate the background to the restructuring of the national reimbursement systems, 2) describe each country’s approaches to engage its public and the resulting public preferences-informed decision frameworks, and 3) identify lessons learned.

## Methods

Belgium and New Zealand serve as case study countries as they recently underwent deliberative processes to change their coverage priority setting systems to explicitly account for public preferences. No other countries were approached for this study.

To increase the validity of the results, we used methodological triangulation by applying two research methods to gather data [23]. In a first step, CL performed a literature review of relevant country documents. Search criteria included the broad terms “deliberative process”, “public engagement in coverage decisions” and “reimbursement of pharmaceuticals”. Results of the literature review informed the development of the topic guide for the interviews. In a second step, we elicited through key informant interviews (in English), country information on rationales and processes for identifying public preferences, and approaches to integrating preferences in coverage decisions. We developed a semi-structured topic guide with 12 open-ended questions (see online Annex 1) which were categorized into four sections: reimbursement decision making prior to collecting public preferences; collection of public preferences and values; integration of public preferences and values into coverage decisions; and experiences with the revised reimbursement decision systems to date. We tested the feasibility of completing the questionnaire among colleagues at the Department of Population Medicine at Harvard Medical School and Harvard Pilgrim Healthcare Institute.

We invited all relevant experts who led the deliberative process on public preferences in each country. Recruitment of interview partners started with outreach to known country health insurance policy experts in Belgium and in New Zealand who recommended topic experts to be interviewed. We interviewed the topic experts who led the deliberative process on public preferences in each country: the three interviewees from Belgium represented the National Institute for Health Insurance (RIZIV/INAMI), the Belgium Health Care Knowledge Center (KCE) and King Baudouin Foundation; the two interviewees from New Zealand represented the Pharmaceutical Management Agency (PHARMAC). Justification of the small sample size is based on the concept of *information power* [24, 25] as the five interview partners were the only experts who could provide in-depth information and experience about the intervention under investigation. In more general terms, qualitative research is intended to elicit views and perspectives from key informants (information rich individuals) which may mean including “biased” individuals to learn about their views. This therefore means that a non-randomized sample is not a limitation of a qualitative study [26, 27].

Interviews were conducted in English by phone between January and February 2017, lasted 1 hour each, and were audio-taped. Participation in the interview was voluntary and consent was implied. In a final step, we reviewed grey literature including government reports of each country, national agency websites and other relevant documents recommended by the interview partners (note: all materials were available in English).

CL and AKW jointly conducted all interviews; recordings were transcribed and reviewed to identify themes and categories of topics mentioned by interviewees [28, 29]. CL and AKW independently read and reread the transcripts and documented emerging themes. After several rounds of review, no new themes emerged. Results were presented and described following the framework suggested by Baltussen et al. [20] Interview partners verified the accuracy of quotes and of the summary. The research protocol was exempt from Human Subject Review and was approved by the Harvard Pilgrim Health Care Institutional Review Board.

## Results

### Reasons for change: outdated reimbursement criteria and changing societal expectations

Representatives of both systems stated that outdated reimbursement criteria that did not explicitly consider patients’ or societal preferences were the main reason for updating coverage processes. In Belgium, the reimbursement process originated in the Law on Compulsory Health Insurance from 1994 [30]. Belgium faced the problem that the general reimbursement process, which required companies to submit full pharmaco-economic analyses for all drugs (including hospital-administered drugs), resulted in delays of up to 1 year in patient access to new medications including those for orphan diseases as pharmaceutical companies had little financial incentive to apply for reimbursement in Belgium.

In New Zealand, PHARMAC’s legal funding obligations were defined in the Operating Policies and Procedures (OPP), which include the reimbursement criteria, the so-called “decision criteria framework” and dated back to 1993. In 2006, PHARMAC decided to review its OPP based on public input to move from a more static (less responsive to the needs of the public) to a dynamic funding process [31]. Experts from New Zealand specifically mentioned that the old OPP defined health outcomes too narrowly with a primary focus on the individual patient leaving out aspects of the wider family and society. In addition, societal expectations have changed requiring a “*social license*” for taking challenging coverage decisions; another major factor was the increasing funding scope of the payer.

*“ … so originally PHARMAC was very much involved in the funding of primary medicines in the community … but over the course of 20 odd years that extended into hospital medicines, it extended into vaccines, medical devices and blood products and other things. So, we really needed to have another look at what the appropriateness of the criteria were.” (interviewee # 1 from New Zealand)*

Both countries’ representatives explicitly mentioned that the goals of restructuring their reimbursement systems were not to construct a new system but rather to update existing evaluation criteria, to create new tools, and to integrate new actors in the coverage decisions processes. In both countries, political and executive leadership committed to implementing a multi-year process of changing the coverage process. In Belgium, in 2010 the national public payer RIZIV/INAMI took a political decision to develop a more transparent and explicit process to account for public preferences in coverage decisions by initiating a multi-year evidence-informed deliberative process (details of this process are described in more detail below). In New Zealand, the executive leaders of the national public payer PHARMAC decided in 2012 to review its OPP and to initiate a multi-year public consultation process to update the reimbursement criteria.

### Deliberative processes of public engagement: multi-year commitment of many stakeholders

While both systems undertook public deliberation over several years to re-evaluate criteria for coverage, each system had a different strategy for public outreach as summarized in Table 1.

**Table 1:**
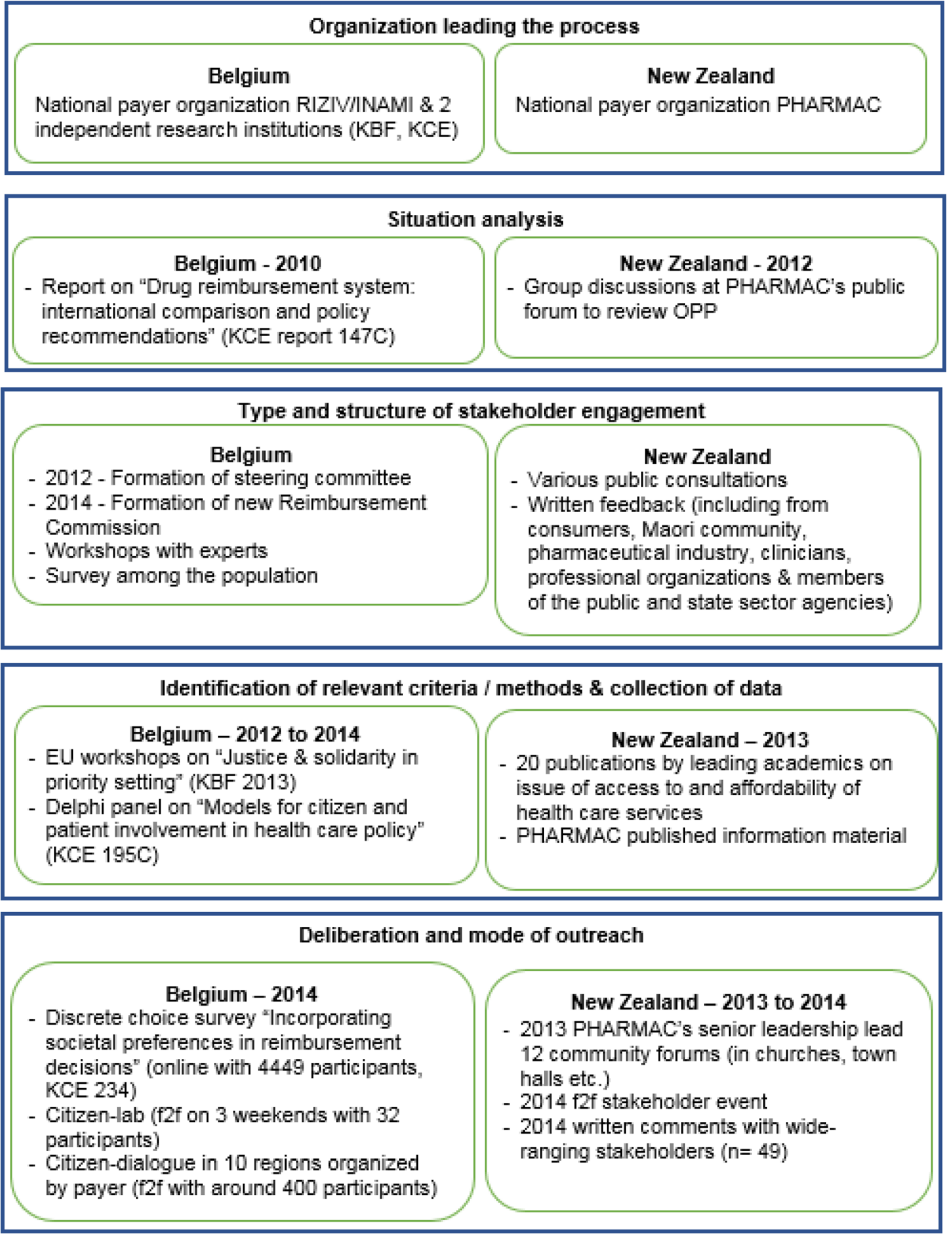
Evidence-informed deliberative processes used to elicit public preferences in Belgium and New Zealand

Abbreviations: *f2f* face-to-face, *KBF* King Baudouin Foundation, *KCE* Belgium Health Care Knowledge Center, *OOP* Operating Policies and Procedures, *PHARMAC* Pharmaceutical Management Agency, *RIZIV/INAMI* Belgium National Institute for Health Insurance

Note: in New Zealand the old funding decision criteria apply to in- and out-patient drugs; the new funding decision criteria apply to in- and out-patient drugs, devices and vaccines. In Belgium, funding decision criteria apply to out-patient drugs

Sources: Evidence-informed deliberative framework modified from [20], KCE report 147C [24], KBF 2013 [33], KCE 195C [34], KCE 234 [35]

Belgium began the process in 2010 with a situation analysis of how other European countries’ coverage decision processes work [32]. The public payer (RIZIV/INAMI) commissioned the semi-governmental research institute Belgium Health Care Knowledge Center (KCE) to compare coverage criteria in Austria, Belgium, France, the Netherlands and Sweden and to evaluate the coverage decision-making processes in each country based on the framework of accountability for reasonableness [5].

*“Well, the aim was really first to do a kind of mapping of what was on the agenda of other institutions in France, in the UK and other countries … [so] we were convinced that we were not the only country, the only actors that were dealing with the same problems, so that it was clear that it was on the agenda of different countries and different institutes.” (interviewee #2 from Belgium)*

They concluded, that to ensure public acceptability of the reimbursement decision criteria, it was essential to be transparent about societal choices. This led to the formation of a steering committee in 2012, including representatives from the national insurer RIZIV/INAMI, researchers, patients and other stakeholders, spearheading the process of eliciting societal values and integrating them in a new reimbursement system.

*“ … the steering committee has played a very important role because we had [persons] from the insurance companies, we had some patients (being the director of a patient platform), we had an independent expert on board, we had the pharma industry around the table … . there was a real engagement right from the start to take it serious.” (interviewee # 3 from Belgium)*

Between December 2012 and December 2014 various methodological workshops, panels and surveys were organized to identify more explicit ways to embed ethical and societal criteria in coverage decision-making. In several workshops, ethicists, health economists and other experts from different European countries developed a conceptual framework for healthcare coverage decision-making including recommendations for stakeholder involvement, structures and processes, assessment criteria, definition of values and objectives as well as barriers and enablers [33]. To define the preferred model for public and patient involvement, a 2-round Delphi survey among a wide range of stakeholders in Belgium was conducted and resulted in the recommendation to involve the public and patients within the existing decision-making bodies, at specific time points, and with changing patient representatives after a period of time [34]. The final part of the multi-year process was the deliberation phase, which consisted of a survey, a “citizen lab” and a citizen- and expert dialogue. A survey (using discrete choice experiments) among the general public (*n* = 4288) and decision makers (*n* = 161) was performed with the goal to generate public preference weights for reimbursement criteria including the therapeutic need, societal need and added value [35]. The citizen lab engaged in debates during 3 weekends with 32 citizen patients, clinical experts and other stakeholders on the question of “Which criteria do citizens consider important in regard to reimbursement of health care, and why?” And finally, the national payer RIZIV/INAMI engaged in a citizen dialogue, which included 10 regional round-table discussions with a total of 400 people, on solidarity and reimbursement of medical costs and other topics (such as overall structure of the national health insurance institution) [36, 37]. The outcomes of Belgium’s process were summarized by KBF (Supplementary Figure 1) and KCE [32] and included an emphasis on quality of life rather than life expectancy, on integrating patient experiences with scientific evidence in the reimbursement process, on involving multidisciplinary teams as part of the reimbursement commission and finally a plea for solidarity.

*“ … when you ask citizens for their opinions as an individual then solidarity appears to become under pressure, but when you give them enough time and the opportunity to discuss these issues among themselves and together with experts and stakeholders then you see the value of solidarity and the right to health for all is very high.” (interviewee # 2 from Belgium)*

In New Zealand, the process of changing the reimbursement criteria began in 2012 with a public forum organized by PHARMAC on the review of its OPP, which were guiding principles for all PHARMAC obligations including procurement, negotiations and public engagement, followed by a round of public consultations. One face-to-face meeting was held and 23 written comments were submitted from a wide range of stakeholders including representatives from the pharmaceutical industry, consumer groups, individual health professionals and professional health groups. This situation analysis resulted in the recommendation to review the pharmaceutical funding application process by first critically assessing the old reimbursement decision criteria [38].

Starting in 2013, PHARMAC led a process of community outreach to consult on the old decision criteria. In preparation for this, PHARMAC prepared a consultation document with background information on the existing decision criteria and possible elements to be included in future decisions [31]. It also commissioned academic publications on coverage decisions for specific diseases like cancer or for defined population groups like people with rare diseases. Overall, PHARMAC’s leaders engaged in 12 public debates in different communities to explain PHARMAC’s role and existing funding structures.

*“ … we ran a very different sort of consultation where we really went out into the community, we went into sort of church halls, village halls … so really an attempt to try and reach out to genuine members of the public and make it sort of as easy for them to get to these forums as we could … ..I think the fact that quite senior management, the chief executive went out to meet the public, we got very, very good feedback. Like it was a good opportunity for us to explain how the system worked because obviously there was some misunderstanding, but really it did give that human face to that decision-making process and actually gave people really the opportunity to ask questions.” (interviewee # 2 from New Zealand)*

With respect to the identification of relevant criteria, PHARMAC decided not to rely on previously established criteria but to let the discussion evolve organically.

*“ … we want to start with a fresh sheet, if you could design it all again … what should we [PHARMAC] take account of and how should we do it?” (interviewee # 1 from New Zealand)*

Following the first round of consultations, PHARMAC integrated all feedback into a new framework of decision criteria, the so-called “factors for consideration”. They then organized a public stakeholder event in April 2014 as well as face-to-face meetings with people who could not attend the community fora and invited written comments. Overall, PHARMAC received 49 written comments [39] from diverse stakeholders including consumer groups, district health boards, clinicians, industry representatives, professional organizations as well as members of the public and state sector agencies. Comments centered on how to weigh criteria as part of coverage decision-making, on the process itself and the involvement of patients and other stakeholders, and the desire to consider in the criteria not only the patient but also the family and the wider society. The public deliberation process also resulted in the initiation of a separate reimbursement process for highly-priced pharmaceuticals for patients with rare diseases [40].

### New reimbursement decision systems: stronger emphasis on quality of life, the separation of individual versus societal perspectives and the importance of “human” decision making

Both countries’ processes of engaging the public to define new reimbursement criteria resulted in a shift towards a greater emphasis on the quality of life of patients’ and their family/caregivers. At the same time both countries concluded that both individual and societal perspectives have to be considered but separately and explicitly as “..*.there is no such thing as a public preference … there is just too much diversity.” (interviewee # 1 from New Zealand)* Experts from Belgium mentioned “… *there is a need to separate questions on own life impact and societal impact and [decision-makers] cannot put those into the same question as most literature does”. (interviewee # 2 from Belgium)* Experts from New Zealand shared that “*… it’s quite a difficult balance to strike, we [PHARMAC] have to be interested in healthcare, we have to be interested in individual and the impact on individuals and at the same time our primary focus is on the system.*” *(interviewee # 1 from New Zealand).*

In addition, both countries concluded that while it is important to explicitly create a list of values, factors to consider and their weights for making coverage decisions on a new pharmaceutical, the final coverage decision still should be “made by a human” who can apply judgment to each situation. As an expert in Belgium put it: “… *the end decision is then taken by the commission as there is still the need for a human to discuss and take the final decision.” (interviewee # 1 from Belgium)* An expert from New Zealand mentioned “… *it became very clear from pretty much all parties that actually it wasn’t desirable to have weighted criteria with some sort of formulaic approach to decision making, they [the public] wanted to feel that given that we were making decisions about patients’ health so they wanted a human guide.* ”*(interviewee # 2 from New Zealand).*

In Belgium, the national payer RIZIV/INAMI set-up a special fund of 10 million Euro for the new pilot reimbursement of the “unmet medical need” program (Supplementary Figure 2), which integrates the public’s and patients’ preferences, with a plan to expand this pilot application to the general reimbursement system including the assessment of medical devices in the future. The pilot reimbursement decision process includes a ranking of high priority unmet medical conditions (in 2018 the list included 62 diseases [30],) and an assessment based on a multi-criteria decisions analysis of the following criteria: medical and societal vulnerability, societal impact as well as the identification and weighting of 19 criteria (including medical-technical aspects, patient perspectives and aspects of solidarity) and six conditions for reimbursement of interventions in health care (Supplementary Figure 2). In addition, the payer established new reimbursement commissions engaging representatives of patients, workers, employers, government, health care providers and the payer. “… *they [the payer] are trying to test there this multidisciplinary approach, they are trying to involve patients, they are looking especially on quality of life”. (interviewee # 1 from Belgium).*

In New Zealand, the factors for consideration were implemented in July 2016 and included four dimensions - need, health benefit, costs / savings and suitability – and a medication’s impact on these is assessed at the individual patient, the family and the wider health system’s level (Supplementary Figure 3). Not every factor might be considered for each coverage decision, but every coverage decision must be taken in relation to available funds within the annual budget.

*“ … we will rank all these applications against each other and we will come up with a list of what’s the next best thing for us to fund, what’s the next best health outcome with the budget that we’ve got.” (interviewee # 1 from Belgium)*

In addition, PHARMAC seeks advice from experts in the Pharmacology and Therapeutics Advisory Board and as a result of the public outreach process has established a mechanism to regularly consult with the consumer advisory board [41].

*“We are doing a lot more of that stakeholder engagement and actually getting out and about with patient groups, so I guess in the past we’ve always engaged with clinicians and government groups but we’re actually sort of engaging a lot more with advocacy groups and stakeholder patient groups and keeping them in the loop more.” (interviewee # 2 from New Zealand)*

### Lessons learned: changing mindsets takes time, engaging the public is enriching for both sides and anticipated challenges of data collection

Both countries’ approaches show that the process of engaging diverse stakeholder groups to revise the reimbursement decision system took several years and demanded openness to input from all involved parties. “*… when you involve citizens or patients … mostly it’s in a kind of open process and you are not sure as initiator what comes out … without knowing where we would land at the end of that process.” (interviewee # 2 from Belgium)* Respondents highlighted the importance of involving all relevant stakeholders from the start of the process to give everyone a voice and sense of being part of the change. This is especially crucial in a system in which health care is organized at federal and state/regional level. As explained by an expert in Belgium:

“*we went also to the Flemish Region and to the Walloon Region to present these results and to look together with them how could we work together on the follow-up, we invited the different ministers and cabinets on our closing conferences and they contributed but they were a little bit irritated that they were not involved right from the beginning.” (interviewee # 3 from Belgium)*

In summary, the processes in both countries were considered a valuable learning experience for everyone and resulted in different perspectives on what should be considered in coverage decisions.

*“ … [this] pilot phase or learning process is going well, actually very well … .it’s changing mindsets, which might be the most important thing, because legislation, criteria and parameters are going to follow afterwards.” (interviewee # 2 from Belgium))*

For the public, the processes were an opportunity to learn about the payer’s operations, how coverage decisions are made and which criteria are considered and gave them a chance to actively shape the future process.

*“ … there is a perception that PHARMAC is all about cost and savings, but actually it’s about health needs and health benefits … and the other misconception was that people thought there were weightings to different decision criteria or different factors, so again by going out it was really a good opportunity to explain that there weren’t weightings. We looked at all factors for all decisions, it wasn’t just all about cost and saving money.” (interviewee # 2 from New Zealand)*

For both payer organizations, the main benefit of the public consultation was to make decision-making processes more transparent and to learn from the public about their preferences.

*“ … for us it [the public outreach] was a huge change and quite a huge amount of work, but I think it has made us ready for the future … we learned a lot through the process how much people value transparency and so we are ensuring that; we’re providing a lot more regular information and updates.”* Another expert mentioned*: “ … we [PHARMAC] did it [the outreach] informally but this gives you that framework to make that decision with all those things clearly explicit.” (interviewee # 1 from New Zealand)*

At the same time, experts from both countries encountered challenges with respect to information needed during the reimbursement decision process on newly included outcome measures such as quality of life of the caregiver. An expert from New Zealand mentioned:

*“ … the data side is actually something we still find quite hard across some of the factors, nobody knows how to get it, so then it is quite difficult to analyze the impacts. But in other areas it’s very straight forward … clinical data … economic evaluation.” “ … what’s been quite hard is just that some of the factors, there just isn’t the literature there available … there are some gaps, but I think that suppliers’ are getting used to what we are asking for, they’ll be putting more information in with their applications.” (interviewee# 1 from New Zealand)*

An expert from Belgium shared that even though some variables might be available, acceptance is not always given. “*It’s very difficult to implement patient-reported outcomes, and patient value and societal value and societal preferences into classic reimbursement decision procedures, because we are so much used to work with very clear algorithms, that number of overall survival ...or stuff like this. We are not used to work with validated judgments.” (interviewee # 2 from Belgium)* Another expert mentioned: “*It is true that even today companies from time to time already come up with quality of life information of patients, [but] they are not easily accepted by the professional decision-makers, [but] now maybe a little bit more.” (interviewee # 1 from Belgium).*

## Discussion

As suggested by Manafò and colleagues [22], there is a great need for a “best practice guide with operational details of public engagement in priority setting”. This qualitative investigation contributes to filling this gap by describing two country examples of steps in evidence-informed deliberative processes for public engagement in priority setting and of implementing public preferences into public drug coverage decisions. The intent of this study was not to judge which country was more effective but rather to describe approaches which may guide other countries’ policy makers seeking to engage the public in coverage decisions.

Despite differences in context and geographic location of the two study countries, our study identified three factors that were crucial for successfully engaging the public to change the national reimbursement system: *1) political commitment is needed to initiate change, 2) broad involvement of stakeholders is required, and 3) commitment of all to engage in a long-term process.*

As described in literature on organizational change theories, “sufficient external environmental pressure such as market or political forces, the existence of internal proponents for the change and the quality of the process undertaken including simple, clearly defined and agreed goals are critical to success.” [42–44] We found that both countries’ processes were initiated by pressures to update coverage processes to better reflect population needs. Interviewees emphasized the need for strong leadership commitment by the national payer organization to lead such a multi-year public engagement process including substantial financial commitment for research funds as well as time of staff but also senior leadership of the payer organization.

Comprehensive involvement of all relevant stakeholders from the beginning of the process was considered crucial. The experts specifically pointed to their positive experiences of broad community outreach but at the same time reflected that they wished to have involved all regions and local community leaders more comprehensively from the beginning of the outreach activity. Typically, stakeholders are described as “any group or individual who can affect or are affected by the achievement of the organization’s objectives” [45]. The healthcare literature acknowledges the importance of stakeholders’ contributions to policy decisions and their beneficial role in changing current policies. It has been shown that early involvement of stakeholders leads to a sense of commitment, common values, trust, and satisfaction with changes [22].

Previous literature pointed to the fact that context, geographic location and cultural differences have to be taken into account when shaping pharmaceutical policies [2]. The observations in this study confirm this notion as Belgium, a country in the heart of Europe, invested in a comprehensive outreach to its neighboring countries at the beginning of their public engagement process to legitimize their approach. On the other hand, New Zealand, an island in the southwestern Pacific Ocean, did not consult with other countries on their public engagement process. In addition, the results showed that cultural differences may lead to differences in how reimbursement processes are shaped. The deliberative process in New Zealand led to a greater emphasis on family and the wider society in the new reimbursement process, while in Belgium it resulted in a greater emphasis on the quality of life of the patient and his/her family.

Finally, as the need for integrating public preferences in policy decision-making is growing, we see a great need to further develop research on public engagement in policy decisions. Policy makers and researchers together should work on developing guidelines and best practice examples that could guide implementation processes of public engagement in pharmaceutical coverage policy decision making. Future research within countries could include an evaluation of different stakeholders’ assessments of the newly revised reimbursement system after several years of implementation. Additional research could compare reimbursement decision processes and outcomes for specific medications in Belgium and New Zealand to those of a country that does not explicitly account for public preference in its reimbursement system.

## Conclusions

This applied policy study describes approaches taken by two national health systems to engage citizens in medication reimbursement priority setting. Their outreach to successfully engage the public, political commitment, broad involvement of stakeholders, consideration of the context and the need for evaluation could guide other countries that consider public engagement processes.

## Supplementary information

**Additional file 1: Figure S1.** Summary of Belgium’s approach to integrating public and patients’ preferences in national reimbursement decisions. Source [37].

**Additional file 2: Figure S2.** Belgium’s unmet medical needs program. Source [37].

**Additional file 3: Figure S3.** New Zealand’s considered factors for drug coverage after re-evaluating the coverage process by involving public. Source: https://www.pharmac.govt.nz/medicines/how-medicines-are-funded/factors-for-consideration/supporting-information/.

**Additional file 4: Online Annex 1.** Interview guide.

## Data Availability

Aggregated data from the interviews are presented in the study.

## Abbreviations

KCE: Belgium Health Care Knowledge Center
OPP: Operating Policies and Procedures
PHARMAC: Pharmaceutical Management Agency (New Zealand)
RIZIV/INAMI: National Institute for Health Insurance (Belgium)
UK: United Kingdom
