# Supplementary figures and images for: Integrating public preferences into national reimbursement decisions: a descriptive comparison of approaches in Belgium and New Zealand
Source: BMC Health Serv Res. 2020 Apr 25;20:351. doi: 10.1186/s12913-020-05152-2 (PMC7183657; doi:10.1186/s12913-020-05152-2)

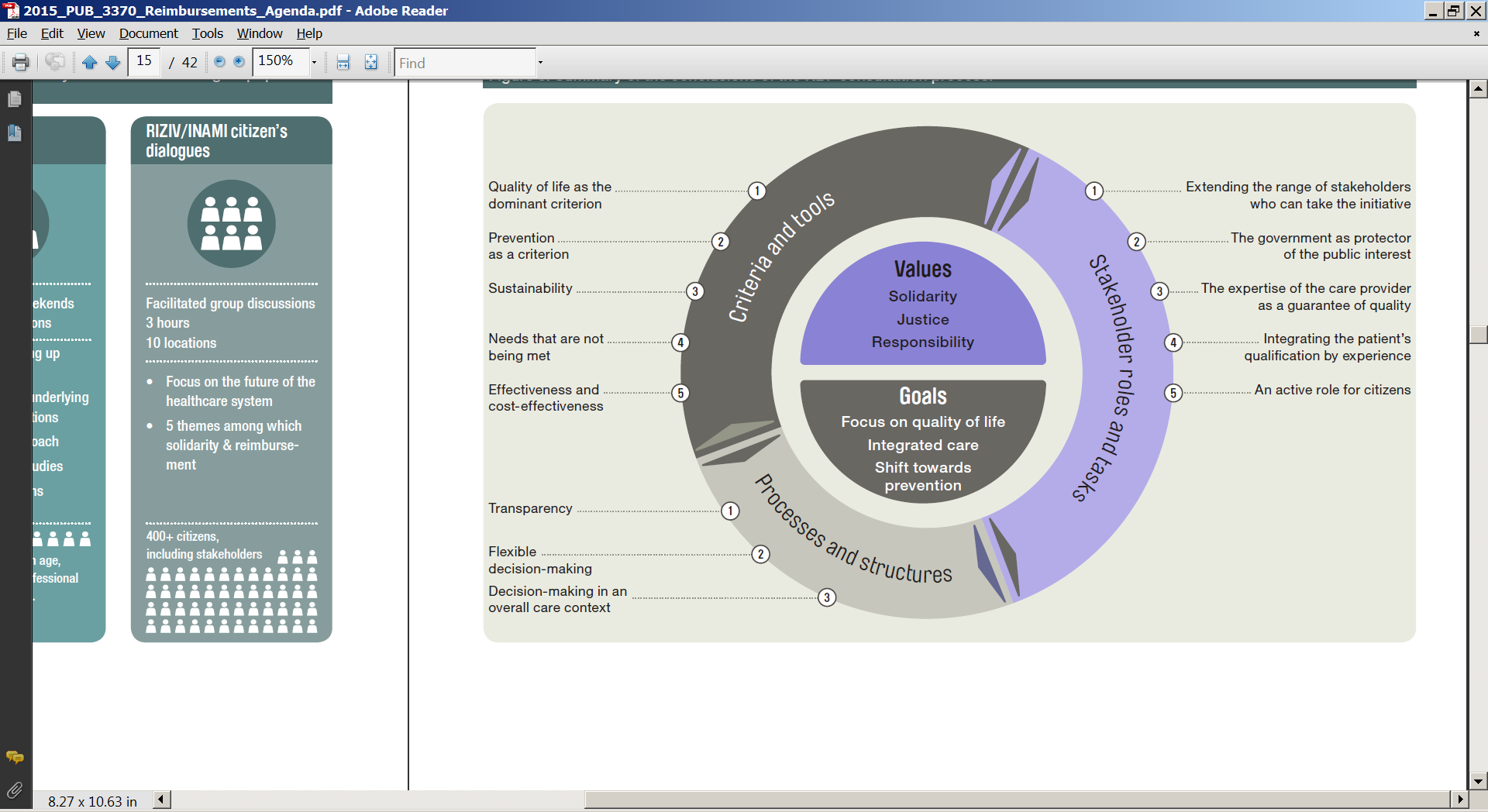

Supplement: Supplementary file 1 — Additional file 1: Figure S1. Summary of Belgium’s approach to integrating public and patients’ preferences in national reimbursement decisions. Source [37]. [file 12913_2020_5152_MOESM1_ESM.docx]

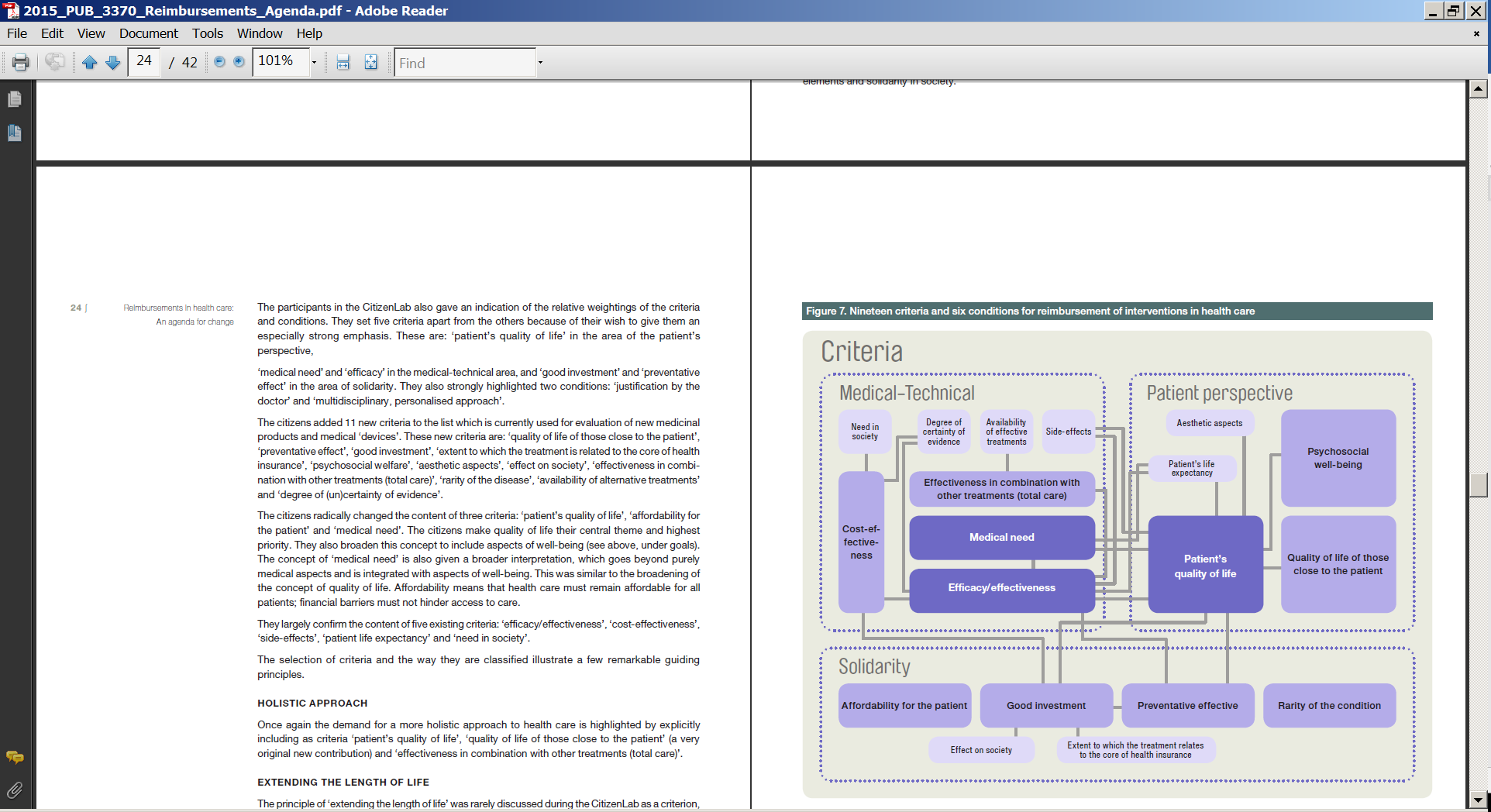

Supplement: Supplementary file 2 — Additional file 2: Figure S2. Belgium’s unmet medical needs program. Source [37]. [file 12913_2020_5152_MOESM2_ESM.docx]

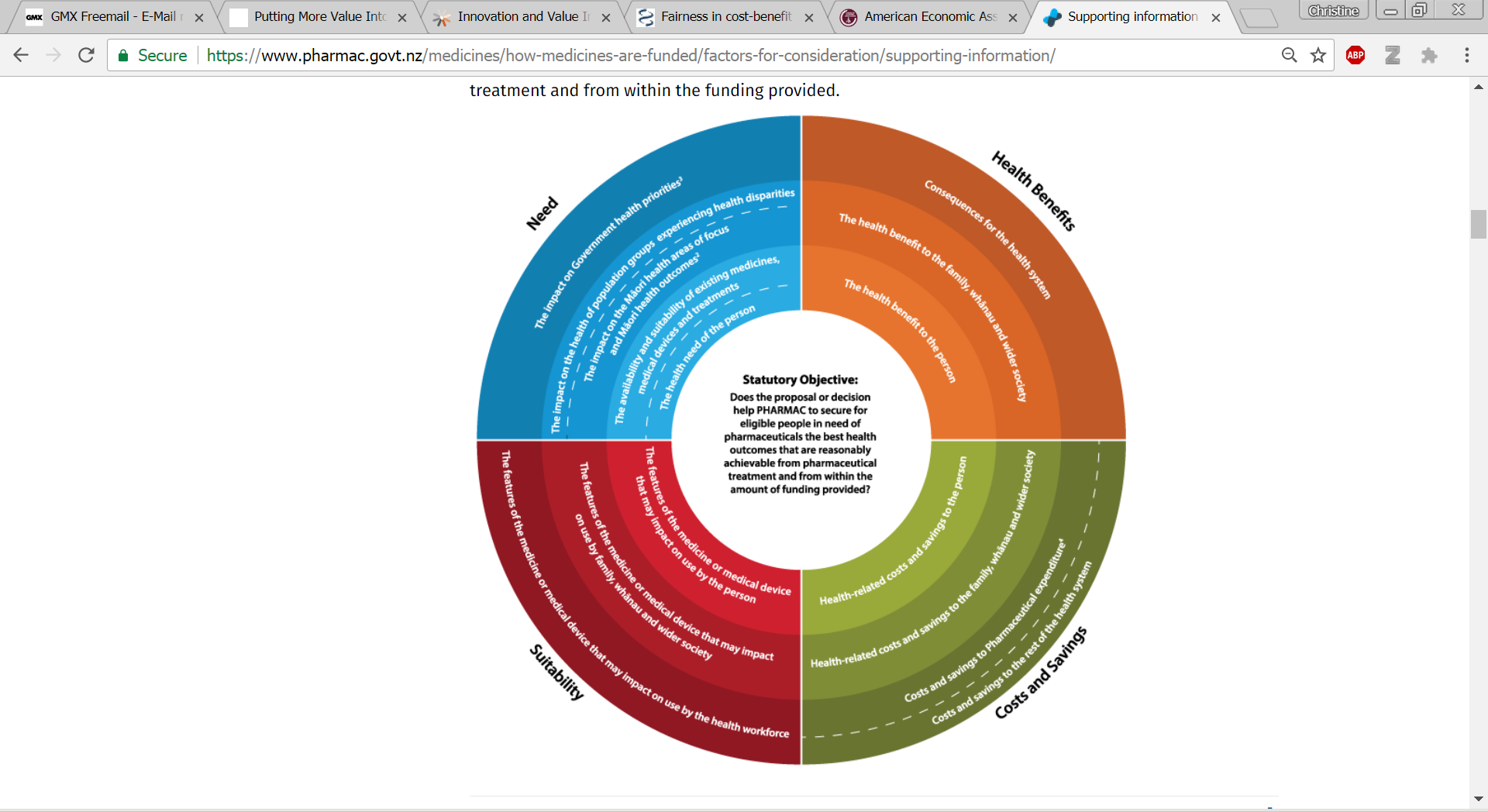

Supplement: Supplementary file 3 — Additional file 3: Figure S3. New Zealand’s considered factors for drug coverage after re-evaluating the coverage process by involving public. Source: https://www.pharmac.govt.nz/medicines/how-medicines-are-funded/factors-for-consideration/supporting-information/. [file 12913_2020_5152_MOESM3_ESM.docx]
