# Supplementary material for: Integrating public preferences into national reimbursement decisions: a descriptive comparison of approaches in Belgium and New Zealand
Source: BMC Health Serv Res. 2020 Apr 25;20:351. doi: 10.1186/s12913-020-05152-2 (PMC7183657; doi:10.1186/s12913-020-05152-2)
Supplement: Supplementary file 4 — Additional file 4: Online Annex 1. Interview guide. [file 12913_2020_5152_MOESM4_ESM.docx]

**Online Annex 1: Interview guide**

**Introduction to the interview**

We thank you for your time and willingness to share your expertise with us today. As explained in our invitation, we are interested in learning about how New Zealand integrated public values and preferences into coverage decisions.

The overall goal of our interviews is to summarize country experiences in changing the pharmaceutical reimbursement system by accounting for public values in coverage decisions. Based on the interview with you and those with other experts, we will summarize approaches to and experiences with the process of preference collection and the integration of those into decision-making. We hope that these experiences will guide similar processes in other countries and systems.

This interview will not take longer than 60 minutes. To ensure accuracy of our summary of responses from each interview, we would like to audio-record our conversation. This recording will allow us to review and correctly summarize the information that you mention. Information from the interviews will be summarized without identifying interviewees.

Some responses may make important points, which we may want to highlight specifically but we will **not** make any references to your person. Do we have your permission to proceed with the interview and audio record this conversation?

- Yes, continue with audio
- No, continue without audio (it may take us a bit longer to take notes during the call.)

Do you have any questions before we proceed?

**Overview of the interview**

We greatly appreciate if you could respond using examples and share your experiences of what may have worked well and what may not have worked prior, during and after the process of eliciting public values and preferences. For this purpose we have structured the interview guide into four sections:

1. Background information prior to collecting public preferences;
2. Data collection of public preferences and values;
3. Data integration into coverage decisions;
4. Follow-up information.

**Background information prior to collecting public preferences**

1. How was the drug assessment and funding process prior to accounting for public preferences? What changed?
2. What prompted the change?
3. Who took the decision to change the process (Ministry of Health, health insurance,…)?
4. When was the new process implemented?
5. What is the goal/objectives of the new coverage process?

**Data collection of public preferences and values**

1. Please tell us about how public preferences and values were elicited?
2. What types of decisions were you seeking public input on? Please give examples.
3. What worked well/didn’t work well during the collection of public preferences and values?

**Data integration into coverage decisions**

1. Please explain the process by which public preferences and values were incorporated into coverage decisions.
2. In case of competing preferences or values, how were these reflected in the coverage process? Please give an example.
3. In the case where public preferences and values might be at odds with other decision factors, how was this addressed? Please give an example.
4. Have there been any coverage decisions since the new system based on public preferences is in place?

**Follow-up information**

1. How were the changes in coverage decisions reported to the public?
2. Please describe the public response to these decisions. Other stakeholder reactions?
3. How successful was the new process in achieving its intended goals/objectives?
4. Based on your experience, what would you continue to do/do differently in the future? Why?
